# A synthetic control study of the effects of comprehensive background check policies on non-fatal firearm crime in five states

**DOI:** 10.1186/s40621-026-00662-9

**Published:** 2026-02-16

**Authors:** Amanda Charbonneau, Christopher McCort, Alex Kwong, Alexander D. McCourt, Daniel Webster, Jon S. Vernick, Garen Wintemute, Rose Kagawa

**Affiliations:** 1 RAND, 1776 Main Street, Santa Monica, CA 90401 USA; 2 Centers for Violence Prevention, Department of Emergency Medicine / VPRP, University of California, Davis, 4150 V Street, Suite 2100, Sacramento, CA 95817 USA; 3 Department of Health Policy and Management, Johns Hopkins University, 624 N. Broadway, Hampton House 580, Baltimore, MD 21205 USA

**Keywords:** Firearm crime, Firearm policy, Comprehensive background checks

## Abstract

**Background:**

Research on the effectiveness of comprehensive background check requirements for firearm purchases often finds individual-level associations that do not translate to the population level. However, few studies assess the effects of these policies on nonfatal firearm violence.

**Methods:**

First, several imputation strategies were compared to mitigate the effects of systematic missingness of crime data reported to the Federal Bureau of Investigation by law enforcement agencies. Next, the imputed data and the augmented synthetic control method, adapted for multiple treated units, were used to estimate the effects of implementing comprehensive background check policies on nonfatal firearm violence in five states. The study period began 15 years prior to the enactment date for each state and extended through 2020.

**Results:**

The results were largely null across multiple specifications and sensitivity analyses.

**Conclusions:**

Background checks are central to many firearm policies in the United States, yet several studies, including this one, do not detect an association between comprehensive background check policies and state-level firearm violence rates. These findings highlight the need to understand how the design, implementation, and enforcement of these laws can be improved.

**Supplementary Information:**

The online version contains supplementary material available at 10.1186/s40621-026-00662-9.

## Background

Federal law [1] requires licensed firearm dealers to run background checks on potential firearm purchasers, with the goal of preventing or deterring purchases by individuals who are legally prohibited from owning or acquiring guns. Such prohibitions can arise from felony convictions, misdemeanor domestic violence convictions, extreme risk or other types of protective orders, or mental illness or substance use disorder adjudications, among other criminal and legal actions.

Firearm transfers between private parties are exempt from the federal background check requirement. There are no reliable measures or proxies for these types of firearm transfers, but survey-based estimates suggest that 28–43% of firearms are acquired from sources other than federally licensed dealers [2]. To address this limitation, some states have expanded background check requirements to all firearms transfers. Known as “comprehensive” or “universal” background check (CBC) policies, these state laws aim to increase the potential effectiveness of the background check system to prevent firearm-related harm.

Findings are mixed from studies that examine the impacts of CBC policies. Four studies using longitudinal panel data to estimate effects for a variety of firearm policies found that comprehensive background check policies were associated with lower rates of homicide [3–6]. One of these studies found this association was only present for African Americans, while another study found the association did not differ for African American and white residents [3, 4]. Yet other studies did not find a protective association [11, 12]. Studies estimating the effects one state at a time (i.e., in California, Colorado, Delaware, Indiana, Tennessee, Oregon, Washington), of implementing or repealing comprehensive background check requirements at the point-of-purchase have typically failed to observe changes in the rates of firearm homicide following policy change [7–10]. One plausible explanation for these findings is that purchasing behavior changed little following policy implementation, as indicated by studies of background check rates in Colorado, Delaware, and Washington [13].

States that require background checks be completed in order to obtain a purchase license, however, have seen significant decreases in their rates of firearm homicide following policy implementation, and significant increases following policy repeal [10, 14, 15]. Studies finding that these laws are associated with reductions in homicide and suicide have hypothesized that the rigor of the licensure process may contribute to the protective effects.

The evidence thus far highlights many of the challenges of estimating the effects of point-of-purchase comprehensive background check laws on rates of firearm violence. Most studies have focused on firearm fatalities, which are rare events in statistical terms. Combined with a limited number of available treated and control units and a treatment that applies to fewer than half of firearm transfers and does not impact firearms already in possession, the chances of generating inconclusive evidence are high.

The current study is novel in its examination of nonfatal firearm crimes, which are more common than firearm homicides [16]. Additionally, the circumstances and victim characteristics of nonfatal firearm violence differ from those of fatal firearm violence, and it is possible CBC policies have greater influence on some forms of violence than others [17]. Specifically, this study estimates the effect of the implementation of comprehensive background check policies on state-level rates of robbery with a firearm and assault with a firearm, relative to expected rates, in Colorado, Delaware, Oregon, Vermont, and Washington.

## Methods

This is a state-level analysis of policy change. Treated states include all those having enacted comprehensive background check policies in the previous ten years (Colorado (2013), Delaware (2013), Oregon (2015), Vermont (2018), Washington (2014)). The study period begins 15 years prior to the enactment date for each state and extends through 2020. Nevada began enforcing its law in 2019 and New Mexico’s took effect in July of 2019. Neither were included as treated states in analyses due to their recent enactment, leaving too few years of follow-up data to estimate effects. 

Donor pool states (those eligible to serve as controls) were all states without a CBC policy for the entire study period. Eligible states included Alabama, Alaska, Arizona, Arkansas, Florida, Georgia, Idaho, Indiana, Kansas, Kentucky, Louisiana, Maine, Minnesota, Mississippi, Missouri, Montana, New Hampshire, North Dakota, Ohio, Oklahoma, South Carolina, South Dakota, Texas, Tennessee, Utah, Virginia, West Virginia, Wisconsin, and Wyoming. States with purchaser licensing policies were not eligible to serve as controls. Missouri repealed its purchase license policy in 2007 and as such, qualifies only for the donor pool for Vermont in the sensitivity analysis with a 10-year pre-intervention period.

### Data

#### Outcomes

The analysis focused on two types of crimes: aggravated assault with a firearm and robbery with a firearm as defined by the FBI’s Uniform Crime Reporting System (UCR) [18]. To summarize those definitions, aggravated assault is an unlawful attack for the purpose of inflicting severe or aggravated bodily injury, usually accompanied by the use of a weapon or means likely to produce death or great bodily harm. Robbery is defined as the taking or attempting to take anything of value from a person by force or threat of force or violence or by putting the victim in fear. The categories of armed robbery include incidents commonly referred to as stickups, hijackings, holdups, heists, carjackings, etc. For both crime types, the number of incidents known to involve a firearm was divided by the state population size to create a rate per 100,000 residents.

CBC policies would not be expected to affect non-firearm crimes. As such, aggravated assault with a knife and non-firearm robbery were incorporated into the analysis as negative controls. These outcomes were chosen due to their similarity in the mean and standard deviation with the main outcomes. If changes were observed in the negative controls when none were expected, this would suggest that the estimates were likely subject to unmeasured biases.

Outcomes were measured using data from “Return A” forms provided by the FBI. Return A forms are submitted by local law enforcement agencies to state and federal agencies as part of the UCR. Reporting to the UCR is voluntary and many agencies either do not submit data or do so intermittently, resulting in extensive missing data on the number of crimes known to law enforcement in some jurisdictions [19]. The FBI imputes missing data at the agency level and aggregates crime estimates to the state, regional, and national level for reporting. Several studies have compared the FBI imputation method with others [20, 21]. To select an imputation strategy for this analysis, four imputation methods were compared. These included the method used by the FBI, an adaptation of the FBI method developed by Joseph Targonski [20], multivariate linear imputation, and predictive mean matching. Descriptions of the data cleaning process and the comparison of imputation methods are available in Appendix A and Appendix Figures 1–3. Based on this comparison of strategies, missing data were imputed using the method described in Targonski [20].

The UCR [22] is also subject to other well-known limitations. These data are affected by the hierarchy rule, under which, if multiple offenses are committed during a single incident, only the most serious is reported. As a result, robberies or aggravated assaults committed in the same incident as a homicide will not be reported. Although the National Incident-Based Reporting System (NIBRS) eliminated the hierarchy rule and mitigates other limitations, the data are available beginning in 2011, just two years prior to CBC policy changes in two of the treated states. In 2018, the year of the most recent policy change included in this analysis, NIBRS data covered 36.8% of the U.S. population [23].

#### Covariates

The analysis included multiple control variables, which are summarized in Table 1, to account for their potentially confounding effects on crime rates. These included measures describing state demographics (percentages of the population that were male, age 18–29, African American, Latino or Hispanic) [24], indicators of state socioeconomic status (mean income and percentages of households earning below the poverty line [30], civilian labor force unemployed [26], and population over age 25 with a high school diploma) [27], criminal justice measures (the number of sworn police officers) [31], individuals imprisoned [32, 33], and violent crimes per 100,000 residents [31]. The proportion of suicides that are completed with a firearm was included as a proxy measure for firearm ownership rates [34, 35]. Additionally, a measure of urbanicity (percentage of population living in an urban area) [31], and average alcohol consumption [36], and pre-intervention measures of the outcomes were also included in the synthetic control models. Missing values were imputed for % population with a high school degree or higher (*n* = 32, 4.6%), % population Latino (*n* = 5, 0.7%), % population age 18 to 29 (*n* = 2, 0.3%), sworn officers per 100,000 residents (*n* = 2, 0.3%) and incarceration rates (*n* = 1, 0.1%).

**Table 1:** Covariates

Domain	Measure	Description
Demographics	% Male	Percentage of the population that is male
	% Age 18–29	Percentage of the population aged 18 to 29 years
	% African American	Percentage of the population identifying as African American
	% Latino/Hispanic	Percentage of the population identifying as Latino or Hispanic
Socioeconomic Status	% Below Poverty Line	Percentage of households with income below the federal poverty line
	% Unemployed	Percentage of the civilian labor force that is unemployed
	% High School Diploma or Higher	Percentage of the population aged ≥ 25 years with at least a high school diploma
	Mean Income	Average income level of state residents
Criminal Justice	Sworn Police Officers	Number of sworn police officers per state
	Incarceration Rate	Number of individuals imprisoned
	Violent Crime Rate	Number of violent crimes per 100,000 residents
Firearm Ownership	% Suicides by Firearm	Proportion of suicides completed using a firearm, used as a proxy for firearm ownership
Urbanicity	% Urban Population	Percentage of the population living in urban areas
Alcohol Use	Average Alcohol Consumption	Mean level of alcohol consumption per capita
Pre-intervention Outcomes	Baseline Outcome Measures	Counts of firearm robberies, firearm assaults, non-firearm robberies (control condition), and knife assaults (control condition)

Missing data were imputed for: % population with a high school diploma or higher (*n* = 32, 4.6%), % population Latino or Hispanic (*n* = 5, 0.7%), % population aged 18–29 (*n* = 2, 0.3%), sworn police officers per 100,000 residents (*n* = 2, 0.3%), and incarceration rates (*n* = 1, 0.1%)

## Analytic approach

The primary analysis used augmented synthetic control methods (ASCM) [39] to estimate the effects of CBC policies on rates of robbery with a firearm and aggravated assault with a firearm. ASCM is well suited for evaluating state-level policy interventions when a limited number of units adopt a policy at different points in time and when treated and untreated units exhibit heterogeneous baseline levels and pre-intervention trends.

The synthetic control method (SCM) estimates a counterfactual outcome for each treated state by constructing a weighted combination of untreated states that closely matches the treated state prior to policy implementation [37, 38]. The weights are chosen to minimize differences in pre-intervention outcome trajectories and selected covariates between the treated state and its synthetic control. The post-intervention divergence between the treated state and the synthetic control is interpreted as the estimated effect of the policy. SCM does not rely on assumptions about comparability. Instead, it uses observed data to identify control units that best reproduce the treated states’ pre-policy trends.

Augmented synthetic control extends this framework by combining the weighting-based synthetic control estimator with an outcome regression model to reduce bias arising from imperfect pre-intervention fit [39]. This is particularly useful in applications with noisy outcomes, limited donor pools, or difficulty achieving exact balance on pre-intervention trends. ASCM allows for negative weights on donor units and applies regularization to limit extrapolation beyond the support of the data, improving performance relative to standard SCM when pre-treatment imbalance remains. In this study, ASCM was implemented using ridge outcome regression, and uncertainty was estimated using the jackknife+ procedure [40], a conformal inference method that provides finite-sample uncertainty intervals without relying on parametric or large-sample assumptions. This approach repeatedly refits the model while leaving out one unit at a time and uses the resulting distribution of prediction errors to construct intervals for counterfactual outcomes.

ASCM was prioritized over population comparisons and conventional regression approaches for several reasons. First, firearm crime rates and trends vary substantially across states. Groupings based on states’ characteristics (e.g., region, urbanicity) would not ensure similarity in pre-intervention trajectories and may obscure within-group heterogeneity. Second, ASCM explicitly prioritizes alignment on pre-intervention outcomes, strengthening causal inference by ensuring that treated states are compared to control units with similar historical trends. Third, ASCM is designed for settings with a small number of treated units and staggered policy adoption.

## Sensitivity analyses

Additional analyses tested the sensitivity of the results to changes in the number of pre-intervention years included, the inclusion of different control variables and donor pool states, the way the lagged value of the outcome was measured (e.g., average, three separate pretreatment values of the outcome), the exclusion of states that changed Stand Your Ground or Shall Issue laws during the two years prior to CBC implementation or during the follow-up period, exclusion of 2020 data, and the exclusion of states with substantial missing data on crime outcomes (Mississippi, Kentucky, New Hampshire, Kansas, and Indiana). Given recent research on the importance of covariate weight optimization, which is not implemented in the AugSynth package used for this analysis, results from the traditional Synthetic Control Method are also included [41].

Finally, a regression analysis was conducted following the model selection process described in a RAND publication [42]. This process compared the false positive and false negative rates for four models, each fit with Poisson and negative binomial distributions. These analyses included all the same control variables except for gun availability and the violent crime rate. Post-treatment measures of these two variables may be influenced by the enactment of CBC policies, and in that case, would act as mediators of the effect in these models. These analyses also controlled for the presence of Stand Your Ground and Shall Issue laws, the passage of which may be associated with the passage of other firearm policies, and which are both associated with firearm crime.

## Results

**Fig. 1:**
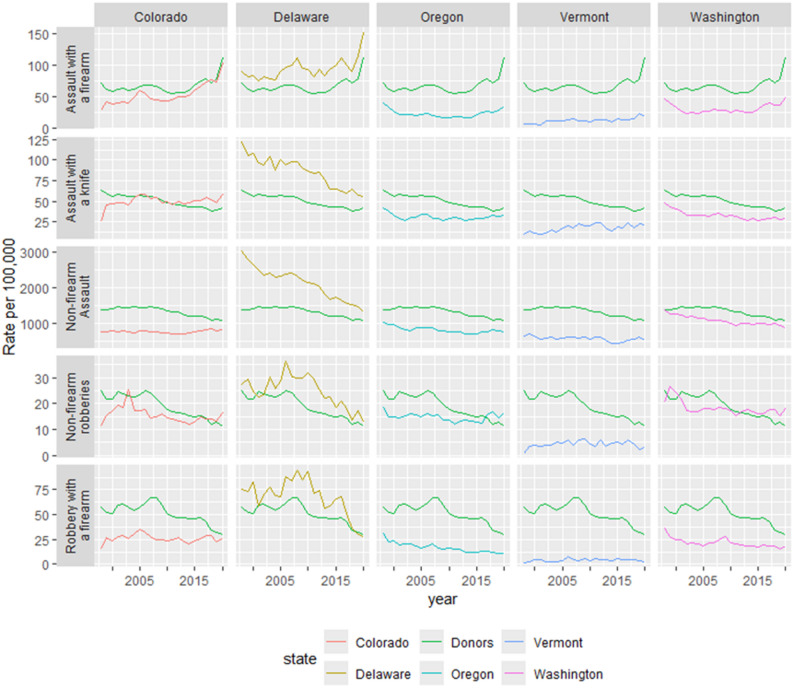
Annual Rates of Firearm Assault, Knife Assault, Non-Firearm Assault, Non-firearm Robbery, and Firearm Robbery by State

Figure 1 compares trends over time in the rates of the four outcomes for each treated state to the average rates among the states eligible for the donor pool. The outcomes are labeled on the y-axes and the donor pool is represented by the green lines. In some cases (e.g., firearm assaults in Colorado), the rates and trends over time are similar for the donor pool and treated state. For others, there are large differences (e.g., knife assaults in Vermont).

Table 2 summarizes the estimated differences in the rates of each outcome in each CBC state individually and as an averaged group. Relative to the variance in state-level rates of firearm robberies and assaults, none of the effects estimated by the ASCM models were large enough in magnitude to suggest a difference between the treated states and the synthetic control following policy implementation. The same was true for non-firearm robberies and for knife assaults, the negative controls. There was also heterogeneity in the direction of the estimated effects for all four outcomes (Table 2). Results were qualitatively similar when each state was modeled separately (Appendix Table 1).

**Table 2:** Estimated effects of CBC policies on Non-Fatal crimes and model fit

	Firearm robberies		Non-firearm robberies		Firearm assaults		Knife assaults	
	ATT (95% CI)	RMSE	ATT (95% CI)	RMSE	ATT (95% CI)	RMSE	ATT (95% CI)	RMSE
Average	0.5 (−9.7, 8.4)	1.5	−0.9 (−5.3, 3.3)	0.8	0.4 (−8.7, 10.9)	1.7	−1.6 (−14.7, 12.9)	2.1
Colorado	2.0 (−6, 10.1)	5.3	−0.5 (−6, 5.6)	2.7	5.6 (−15, 23.5)	6.1	−0.4 (−14.1, 10.5)	6.1
Delaware	0.1 (−14.5, 16.9)	9.2	−5.2 (−19.4, 8.3)	3.1	1.9 (−15.1, 19.4)	6	−13.5 (−71.1, 53.9)	5.8
Oregon	−0.5 (−10.4, 8.9)	2.7	1.8 (−5.3, 9.4)	1.3	−5.1 (−25.7, 19.8)	3.8	2.4 (−9.7, 12.9)	3.4
Vermont	2.4 (−30.2, 27.5)	1.5	0.03 (−7.8, 7.2)	1.3	1.0 (−29.4, 33.9)	2.7	3.0 (−22.5, 23.6)	3.3
Washington	−1.4 (−11.2, 7)	3.2	−0.7 (−4.6, 3.8)	2.7	−1.6 (−13.5, 11.5)	4.4	0.3 (−8.6, 7.3)	3.9

ATT=Average Treatment Effect on the Treated, shown as the number of crimes per 100,000 persons relative to the estimated number of crimes in the synthetic control

**Fig. 2:**
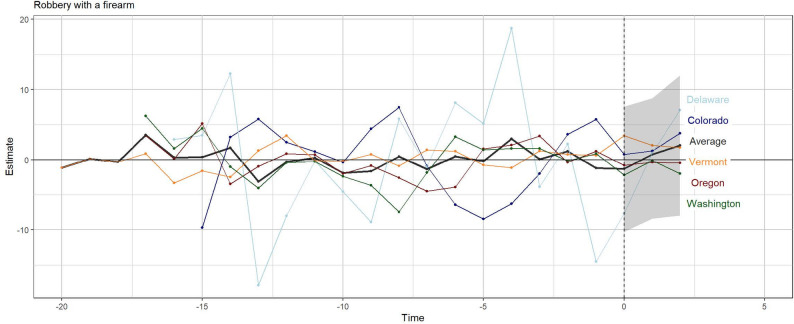
Firearm robberies per 100,000 persons relative to the synthetic control

**Fig. 3:**
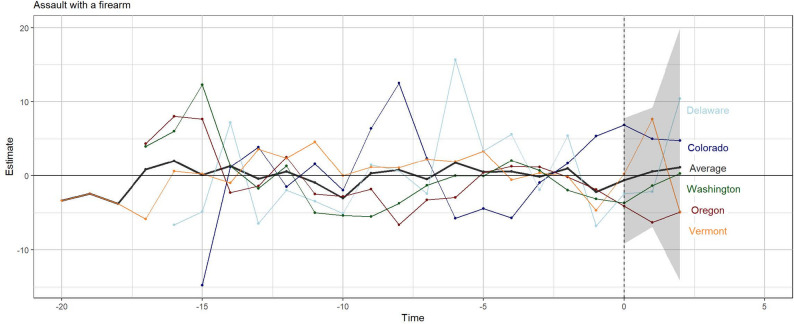
Firearm assaults per 100,000 persons relative to the synthetic control

The weights applied to the donor pool states are shown in Appendix Figure 4. The average root mean square error (RMSE) was 1.5 for the firearm robbery model (approximately 5% of the mean) and 1.7 for the firearm assault model (roughly 4% of the mean) (Table 2). The lower the RMSE, the better the fit. While there is clear variation around the treated states in the pre-treatment period (Fig. 2 and 3), the RMSEs, relative to the standard deviation of the outcome in each state, are indicative of a moderately good fit for all states except Vermont in the firearm assault model. In this case, the RMSE is nearly double the standard deviation of the outcome. Additionally, the standard deviation of the outcome for Delaware indicates a higher degree of variation, a scenario that makes finding a good fit more challenging. While the RMSE is small relative to this standard deviation, it is elevated relative to the mean.

Figures 2 and 3 depict the results for the two main outcomes. Figure 2 shows the differences in firearm robberies per 100,000 persons between each treated state and its synthetic control over time. The horizontal axis represents time relative to CBC policy implementation, with the dashed vertical line indicating the year of enactment for each state (Time = 0). The vertical axis shows the estimated difference between observed and synthetic outcomes. Although there is variability in the pre-enactment period (Time < 0), particularly for Delaware, the differences do not appear to be systematic (e.g., all positive or negative). Following policy implementation (Time > 0), estimated differences for each state and the average remain centered near zero and within the range of pre-intervention variation. The shaded region indicates uncertainty around the average estimate, which widens in later post-intervention years, reflecting increased uncertainty as fewer post-policy observations are available.

Figure 3 provides a similar gap plot for aggravated assaults with a firearm. While the differences are still relatively large for Delaware, they are not as extreme as those for firearm robberies and there are other states with similar patterns (i.e., Colorado and Washington). Following policy implementation (Time > 0), estimated differences for each state and the average remain centered near zero and within the range of pre-intervention variation. Similar plots for the negative controls are available in Appendix Figures 5 and 6.

### Sensitivity analysis findings

Results across five sensitivity analyses were similar to the results from the main analysis (Appendix Tables 2–8). The first of these added a population covariate to synthetic control models. The second analysis shortened the pre-intervention period to 10 years. In a third analysis, states were dropped from the donor pool states if they implemented stand-your-ground policies during the study period (Idaho, Kansas, Maine, Mississippi, North Dakota, West Virginia, Wisconsin, and Wyoming). In a fourth sensitivity analysis, data from 2020 were excluded. Finally, the states with substantial missing data were excluded (Mississippi, Kentucky, New Hampshire, Kansas, and Indiana). Results were null for all outcomes across all analyses.

In the comparison of false positive and false negative rates generated from four traditional regression models, the best performing model was a negative binomial with change coded effects and an auto-regressive lag term for each state (Appendix B); results are presented for this model in Appendix Table 7.

Appendix Figure 7 shows the proportion of simulations for which the p-value was less than 0.05. This would be expected to occur in 5% of simulations under the null, but the proportion is greater for all models, suggesting that the tested models would lead to false positives more often than the standard threshold for such errors. With that caveat, the coefficient for Vermont’s policy change was statistically significant and negative for firearm robberies. However, the coefficient was similarly negative and statically significant for the negative control, non-firearm robberies.

## Discussion

Analyses using augmented synthetic control techniques and negative binomial regression models indicated uncertain and heterogeneous effects of expanding point-of-sale background check requirements on non-fatal firearm crime rates. The augmented synthetic control analysis suggested that the implementation of comprehensive background checks was associated with small changes in firearm robberies and firearm assaults that were not well distinguished from zero. Results from multiple specifications of the ASCM models and the negative binomial model were similar.

These findings align with those of several other state-level studies of comprehensive background check policies, which have typically investigated effects on firearm homicides and suicides [7–10, 43]. These prior studies also did not observe changes in firearm homicide rates after the implementation or repeal of comprehensive background check requirements at the state level. This study extends this body of research to non-fatal firearm crimes, which occur with much greater frequency [16].

The primary motivation for conducting this study was to identify whether the effects of CBC implementation are better identified for a less rare outcome (nonfatal firearm violence vs. fatal firearm violence). While less rare, these outcomes appeared to be more variable, making it difficult to distinguish patterns from noise.

In addition to statistical explanations, this null finding leaves open many avenues of interpretation. First, comprehensive background check policies as implemented may not be sufficient to yield state-level changes in rates of nonfatal firearm violence. Individual-level studies suggest background checks are effective when they appropriately result in a denial [44, 45]. However, to observe state-level changes requires that individuals at elevated risk for violence have risk factors that correspond to a prohibiting criterion, that records for these risk factors are available during the check, a background check is in fact completed, and the prospective purchaser does not circumvent the system through means such as theft. These factors are not always met. Prior studies observed that individuals who pass background checks and are legally authorized to buy firearms can still go on to commit crimes, evidence that the current prohibiting criteria are imperfect predictors of risk [46]. Additionally, results from the 2019 National Firearm Survey found that 22% of gun owners obtained their most recent firearm without a background check, even in states that require them [2]. Sales to straw purchasers are the second most common source of crime guns and demonstrate that some people find ways to circumvent the system [47]. More generally, the prevalence of firearms in the United States and the existence of an illicit market provide ample opportunity to avoid a background check [48]. Without addressing these gaps in the design and implementation of comprehensive background checks, it may be unreasonable to expect larger reductions in population-level firearm crime rates.

There are several important limitations to consider when interpreting these results. First, state-level analyses may not detect important variation in outcomes among sub-populations. Furthermore, although this study examines crimes that occur more often than firearm homicides, these are still rare events that are not evenly distributed across sub-populations, geography, or time. Ownership of and means of obtaining firearms are also likely to vary across these dimensions. Guns used in violent crimes may be more likely to be obtained illegally and therefore less sensitive to changes in background check policies.

Additionally, UCR crime data reflects only the most serious offense associated with an incident, and these are reported voluntarily by law enforcement agencies. Relative to homicides, robberies and assaults may be subject to reclassification or misclassification, and practices may vary widely between law enforcement agencies. There are few tools available to assess the quality of the data, and missingness is a well-known limitation [20]; at the extreme, more than 60% of the data on firearm assaults was missing for one state. To address missingness, several imputation strategies were tested and an approach similar to the one outlined by Targonski [20] was ultimately implemented.

Even if all law enforcement agencies provide complete data, non-fatal firearm crimes could still be undercounted due to incidents going unreported by victims. However, underreporting varies depending on the crime type, and violent crimes are less likely to go unreported compared to other types of crimes. For instance, approximately 72.8% of firearm victimizations are reported to the police, while 30.8% of property crimes are reported to the police [49]. Bias in the study may also be present if the level of underreporting varies between states. Although the magnitude of this variation is unknown, a previous study identified variation in crime reporting between urban areas in the Northeast and rural areas in the Midwest [50].

This study’s methodological limitations include the small number of states. This is a common issue f [7]or state-level synthetic control analyses [8–10, 43] that is only partially mitigated by accounting for staggered implementation dates and then pooling results [51]. In addition to the limited number of treated units, the donor pool was also constrained to 32 states; in practice, only 4 to 8 states were weighted positively in the development of the synthetic “state.”

With respect to confounding, other state-level policy changes may have influenced the results. Excluding states that implemented Stand Yyour Ground or Shall Issue laws did not change the substantive interpretation of the findings. However, other firearm policies (e.g., extreme risk protection orders) or measurement error (e.g., accuracy of the proxy for firearm ownership) could also affect the results.

Future research on background check policies would benefit from deeper explorations of processes and mechanisms. For example, it would be important to investigate whether potential buyers and sellers are aware of the background check requirements associated with private party transfers. Future studies could also explore any differences in compliance rates between private party transfers and dealer transactions.

## Conclusion

Background check policies are associated with several challenges related to implementation and enforcement, and it could be even more difficult to enforce extensions of the requirement to private-party firearm transfers, especially in the absence of a permit-based system. The quality of the data on prohibiting factors has improved over time, which should increase the likelihood that a person who meets prohibiting criteria will be prevented from purchasing a firearm *if* a background check is completed. However, it is unclear how many perpetrators of firearm crimes would not or could not gain access to a firearm without a background check.

Although there is some evidence that background checks conducted by licensed firearm dealers lead to reductions in firearm homicides, it is unclear whether extending background check requirements to private transfers–in the absence of permitting requirements–leads to additional reductions. Far less is known about the effects of these policies on non-fatal firearm crimes. Although these findings do not support strong conclusions, this study extends the breadth of outcomes under consideration, and explores imputation strategies and model selection processes that could inform efforts to address the challenges associated with historical crime data.

## Supplementary Information

Supplementary Material

## Data Availability

The datasets generated and/or analyzed during the current study are available from the corresponding author on reasonable request.

## Abbreviations

ASCM: Augmented Synthetic Control Method
CBC: Comprehensive background check
RMSE: Root Mean Squared Error
SCM: Synthetic Control Method
