# Supplementary material for: A synthetic control study of the effects of comprehensive background check policies on non-fatal firearm crime in five states
Source: Inj Epidemiol. 2026 Feb 16;13:21. doi: 10.1186/s40621-026-00662-9 (PMC13011692; doi:10.1186/s40621-026-00662-9)
Supplement: Supplementary file 1 — Supplementary Material [file 40621_2026_662_MOESM1_ESM.docx]

A Synthetic Control Study of the Effects of Comprehensive Background Check Policies on Non-Fatal Firearm Crime in Five States

Amanda Charbonneau, Christopher McCort, Alex Kwong, Alexander D. McCourt, Daniel Webster, Jon S. Vernick, Garen Wintemute, Rose Kagawa

**APPENDICES**

Table of Contents

[Appendix A: Crime Data Imputation 3](#_Toc220661862)

[**Appendix Figure 1. Example Aggregation Patterns by Originating Agency Identifier (ORI)** 3](#_Toc220661863)

[**Appendix Figure 2. Missing Data by State** 5](#_Toc220661864)

[**Appendix Figure 3. Missing Data by State and Year** 6](#_Toc220661865)

[Appendix Table 1. Single-State ASCM 8](#_Toc220661866)

[Appendix Figure 4. Multi-State ASCM Donor Weights 9](#_Toc220661867)

[Appendix Figure 5. Non-Firearm Robberies per 100,000 Persons Relative to Synthetic Control 10](#_Toc220661868)

[Appendix Figure 6. Knife Assaults per 100,000 Persons Relative to Synthetic Control 10](#_Toc220661869)

[Appendix Table 2. Multi-State ASCM with Population Included as a Covariate 11](#_Toc220661870)

[Appendix Table 3. Multi-State ASCM with 10 Years Included in the Pre-Intervention Period 12](#_Toc220661871)

[Appendix Table 4. Multi-State ASCM Restricting to States Without Stand Your Ground or Shall Issue Law Changes During Key Periods 13](#_Toc220661872)

[Appendix Table 5. Multi-State ASCM Excluding 2020 Data 14](#_Toc220661873)

[Appendix Table 6. Multi-State ASCM Excluding States with Substantial Missing Data 15](#_Toc220661874)

[Appendix B. Final Regression Model Equation 16](#_Toc220661875)

[Appendix Figure 7. False Discovery Rates for Four Regression Models Tested 17](#_Toc220661876)

[Appendix Table 7. Coefficients and P-Values from the Negative Binomial Regression Model 18](#_Toc220661877)

# **Appendix A: Crime Data Imputation**

Data from Return A files are available at the agency (referred to as Originating Agency Identifiers or ORI) and month levels. Our analyses use data aggregated to the state and year. However, our data cleaning began at the ORI-month level.

Our first step was to distinguish reliable data from data that would need to be imputed. ORIs that reported a non-zero value for the outcome of interest (y) or provided a reason for non-reporting (e.g. data reporting for the ORI was “covered by” another ORI or data from one month were “reported in” another month) in all months and across all study years (1990-2020) were accepted as sufficiently reliable without further editing.

**Aggregation Patterns**

In many cases, ORIs reported crimes at intervals other than monthly (e.g. quarterly, biannually, or annually). In such cases, zeros are reported in some months, and larger crime counts are reported in other months within a given year. These agency-months could be identified by a run of 0 values (without reference to an explanation such as “covered by” or “month reported in”) for the outcome of interest over 1 to 11 months followed by a large non-zero value. In these cases, we hypothesized that ORIs were reporting aggregated crime counts in the non-zero months. If this were true, we would expect the monthly mean of non-aggregate years * 12/n to equal the mean of the potentially aggregated months, where n is the number of months with potential aggregates. For example, an ORI in Rhode Island appeared to report crime counts at quarterly intervals for a span of several years (Appendix Figure 1). We compared the mean of non-zero crime counts for those years to the mean of all crime reports for years not identified as potential aggregates multiplied by three. We arrived at a multiplier of three here because it appeared the data were being reported in quarterly intervals ((12/4)=3).

### **Appendix Figure 1. Example Aggregation Patterns by Originating Agency Identifier (ORI)**


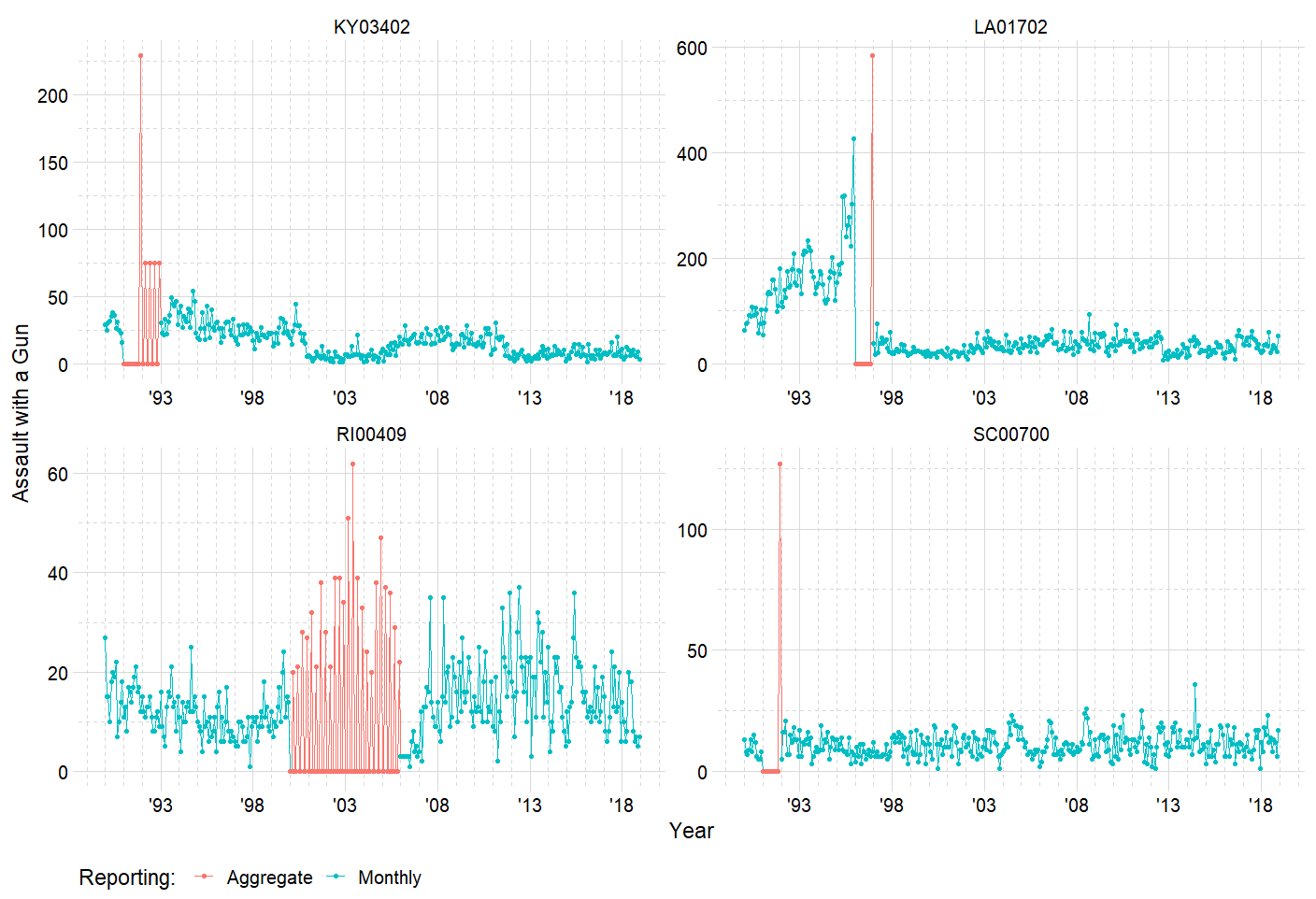


To operationalize this theory, we calculated the ratio of the mean crime count for agency-months in potential aggregate months to the mean count of crimes reported monthly in non-aggregate years. We then compared our calculated ratio to sqrt(12/number of months reported). This comparison value provides a lower threshold in order to account for fluctuations around the expected value. Ratios that exceeded this value were marked as aggregate years.

Some aggregation months were less easily identified. To identify these, we created normalized y values by subtracting the agency mean y (excluding “covered by” months, months with a zero value, and previously identified aggregate months) and dividing by the standard deviation to create z-scores. An observation was marked as an aggregate if it was: 1) preceded by a zero in the same year, 2) not preceded by a record we had already determined to be acceptable in the same year, 3) not already marked as aggregate, and 4) had a z-score greater than 1.96.

We divided crime counts reported in months we identified as aggregate months evenly among the aggregated month(s) and the months with zero values in the same ORI and year. These ORI-months were then included with no additional changes.

A few ORIs had no apparently reliable data that could be used for comparison. However, if an ORI only ever reported non-zero values in a single month of each year, we took these values as aggregates and divided the reported values evenly across ORI-months within years.

**Identifying Missing Values**

Missing data are reported as zeros in the FBI Return A files. Therefore, our next step was to distinguish true zeros from zeros standing in for missing data for those zeros that had not already been replaced by the process described above. To determine if the zeros within an ORI-year occurred more frequently than one would expect given a Poisson random variable with the observed rate, we used a score test for zero inflation (Van den Broek, 1995). We corrected p-values to account for multiple tests using the approach described in Benjamini & Yekutieli (2001). We replaced zeros with corresponding corrected p-values<0.001 with missing values. To identify potentially missing years (i.e. entire years reported as zero, when, in fact, the data were likely missing), the same score test was administered on the ORI-year scale, after summing crime data to the year level, with the same correction and cut-off.

Agencies that never reported values other than zero or reported that their crime reports were “covered by” another ORI did not provide sufficient information for us to find patterns of missingness. These ORIs and their population counts are excluded from the data.

The population value for agencies was also missing at times. For agencies that were missing population values for some months but not others, missing values were interpolated using a linear model. Agencies with all missing population were assigned population 1 to avoid undefined rates in the imputation step described below.

As reported in Targonski (2011) the “date last update” field in the Return A data file indicates the date the FBI received the Return A file. Any remaining zeros in the data that were reported along with an invalid date of last update field (i.e. missing or all zeros) were replaced with missing values.

The resulting data had varying levels of missing data (Appendix Figures 2 and 3). South Carolina had the lowest proportion of missing data (per population-month) while Illinois was an outlier with more than half of the data marked as needing imputation. Appendix Figure 3 shows variation in missingness across years within states.

### **Appendix Figure 2. Missing Data by State**


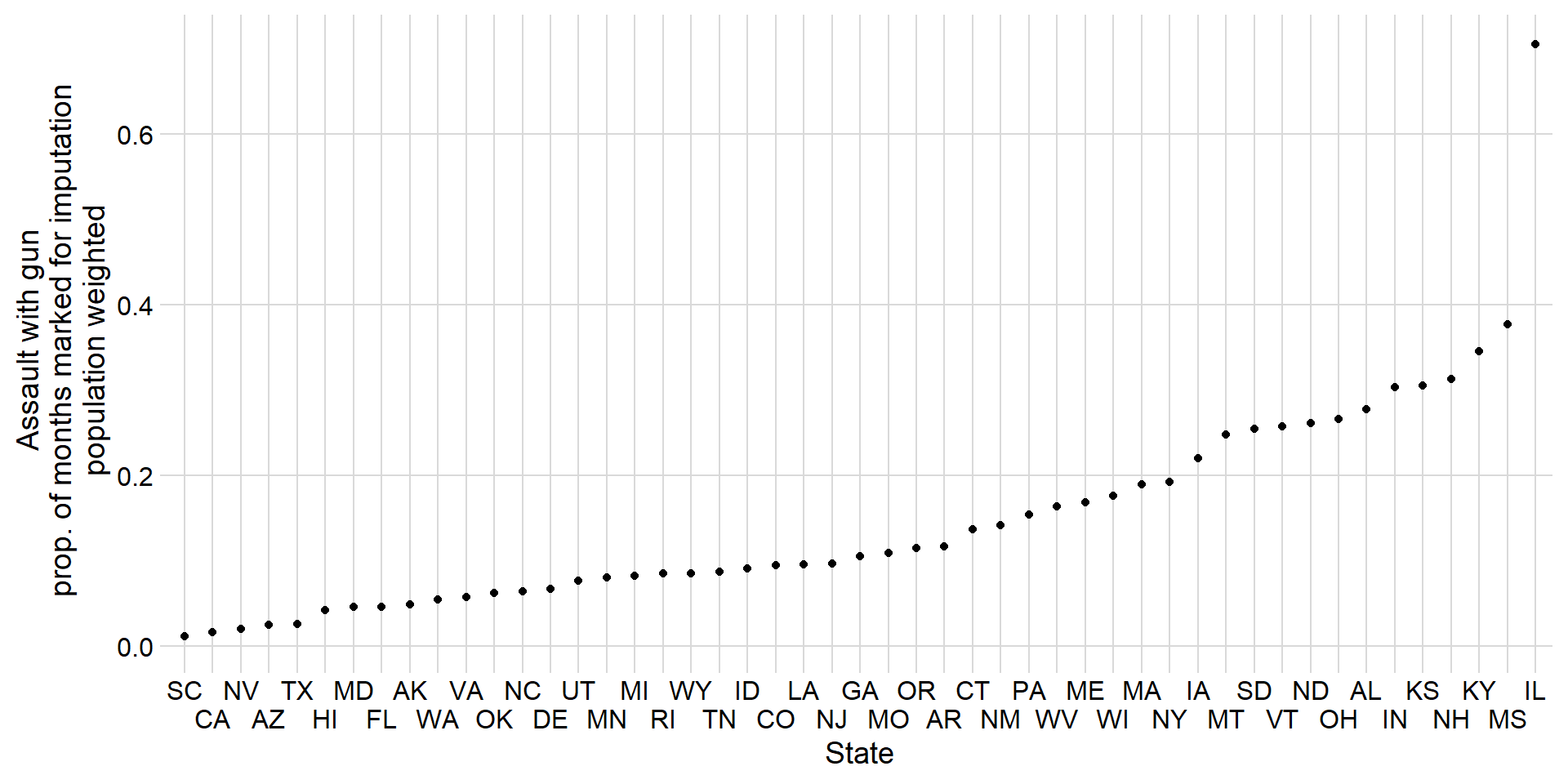


### **Appendix Figure 3. Missing Data by State and Year**


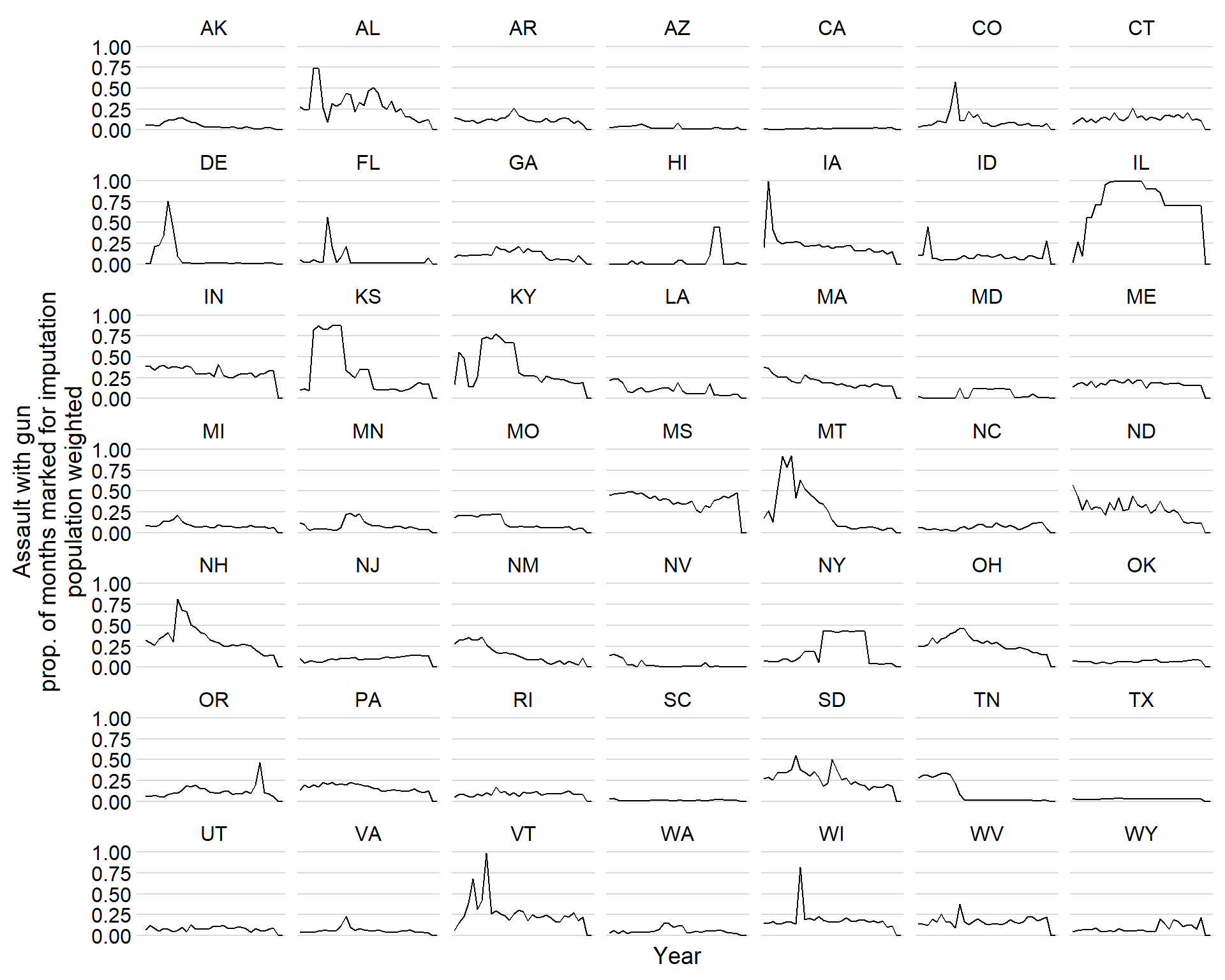


**Imputation**

We compared four imputation strategies in order to determine the one we would implement. These included the method used by the FBI, an adaptation of the FBI method developed by Targonski, multivariate linear imputation, and predictive mean matching. Traditionally, the FBI imputes missing month values at the ORI-level using the average number of crimes reported by that ORI in other months the same year. If an ORI reports data for fewer than three months, however, no reported data are used and instead the value is imputed using the crime rate from agencies in the same state that cover a population similar in size. Targonski extends this method to include data reported the year prior in the same ORI. First, the rate of change from year to year is calculated using only the months with reported data for the index year and the prior year. This rate of change is then applied to the months in the year prior to estimate values for the missing months in the index year. If an ORI is missing more than three months of data, a similar method is applied except the rate of change is determined using ORIs serving similarly sized populations in the same state. This is described in more detail in Targonski (2011). We also applied more standard imputation methods. In our multivariate linear imputation models, we included ORI population, an indicator for ORIs with zero population, and the first three principal components of the counts of offenses not being imputed. Finally, predictive mean matching iteratively generates a set of coefficients from a linear regression model using the observed data, replacing those coefficients with values selected at random from the posterior predictive distribution of the produced coefficients and using these coefficients to predict values for the entire range of the variable of interest (van Buuren & Groothuis-Oudshoorn, 2011). A random selection is chosen from the set of observations with predicted values that are similar to the predicted values for missing observations.

To decide which method to employ, we first took the subset of Return A File data we had determined was sufficiently reliable and knocked out values at random, replicating the observed patterns of missingness. We then used each method to impute the knocked-out values and compared the imputed values to the known values using the mean absolute error and the root mean squared error. The method developed by Targonski performed the best. We returned to the full dataset and imputed all data we had marked as missing in the data cleaning phase of this project using Targonski’s (2011) longitudinal adaptation of the FBI’s method for imputing crime data.

Briefly, for ORIs missing no more than two months of data in a year, we calculated the rate of change in the crime count from year to year within the ORI using observed months. To impute a specific month’s value, we multiplied the crime count value from the same month in the previous year by the year-to-year rate of change calculated from the observed months. For ORIs reporting fewer than 3 months of data in a year, the rate of change is borrowed from the rate of change for similar ORIs (i.e., ORIs in the same state and FBI group, which are defined by population size). We imputed iteratively in time, so that consecutive years needing imputation would rely on the previous complete year.

**Limitations**

Due to the hierarchy rule, in the Uniform Crime Reports, robberies and assaults that occurred within the same incident as a homicide are not reported to the FBI. Only the most serious incident is reported. Similarly, for an incident involving robbery and aggravated assault, only the robbery would be reported. Our imputation method did not address missingness caused by underreporting due to the application of the hierarchy rule. We also did not address missingness due to nonreporting of crimes to police.

*References*

Benjamini, Y and Yekutieli, D. (2001). The Control of the False Discovery Rate in Multiple Testing Under Dependency. The Annals of Statistics, 29(4), 1165-1188. doi: 10.1214/aos/1013699998

Targonski, J. R. (2011). A Comparison of Imputation Methodologies in the Offenses-Known Uniform Crime Reports. <https://www.ojp.gov/pdffiles1/nij/grants/235152.pdf>

van Buuren, S., & Groothuis-Oudshoorn, K. (2011). mice: Multivariate Imputation by Chained Equations in R. Journal of Statistical Software, 45(3), 1–67. <https://doi.org/10.18637/jss.v045.i03>

Van den Broek, J. (1995). A Score Test for Zero Inflation in a Poisson Distribution. Biometrics, 51(2), 738-743. doi:10.2307/2532959

# **Appendix Table 1. Single-State ASCM**

|  | **Colorado**  **ATT (p-value)** | **Delaware**  **ATT (p-value)** | **Oregon**  **ATT (p-value)** | **Vermont**  **ATT (p-value)** | **Washington**  **ATT (p-value)** |
| --- | --- | --- | --- | --- | --- |
| **Robbery with a firearm** | 1.243 (0.912) | 2.091 (0.846) | 1.646 (0.802) | 15.926 (0.304) | 1.399 (0.884) |
| **Non-firearm robberies** | -3.180 (0.249) | 2.373 (0.661) | 0.243 (0.952) | 18.693 (0.252) | -6.411 (0.093) |
| **Assault with a firearm** | 5.246 (0.687) | -0.698 (0.968) | -4.477 (0.541) | -7.191 (0.879) | -10.442 (0.388) |
| **Assault with a knife** | 2.356 (0.749) | -12.035 (0.302) | -1.149 (0.896) | 14.688 (0.438) | -7.853 (0.187) |

ATT=Average treatment effect on the treated, shown as the number of crimes per 100,000 persons relative to the estimated number of crimes in the synthetic control. Estimates are from augmented synthetic control models using one treated state per model. P-value in parentheses are generated using the jackknife procedure.

# **Appendix Figure 4. Multi-State ASCM Donor Weights**


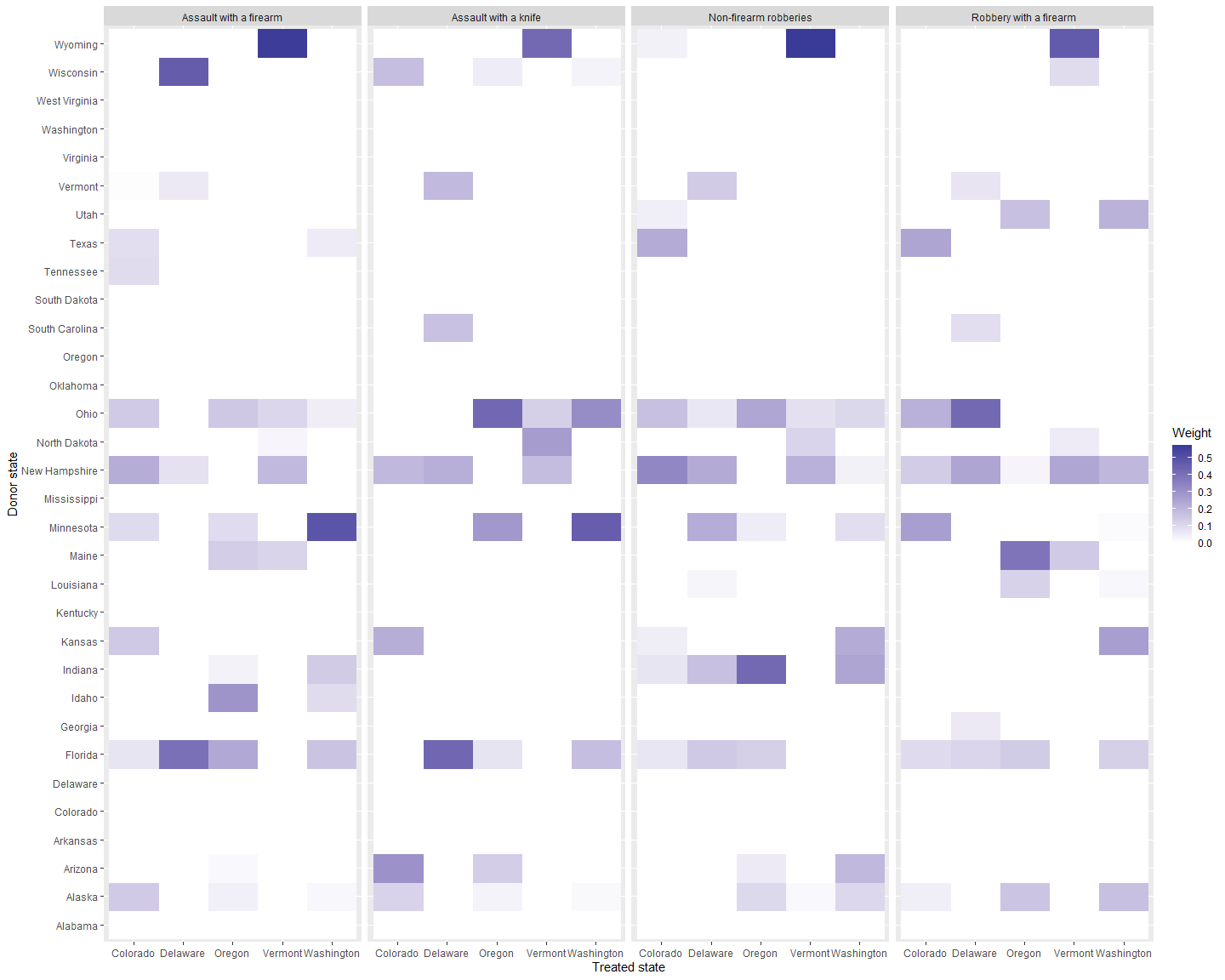


# **Appendix Figure 5. Non-Firearm Robberies per 100,000 Persons Relative to Synthetic Control**


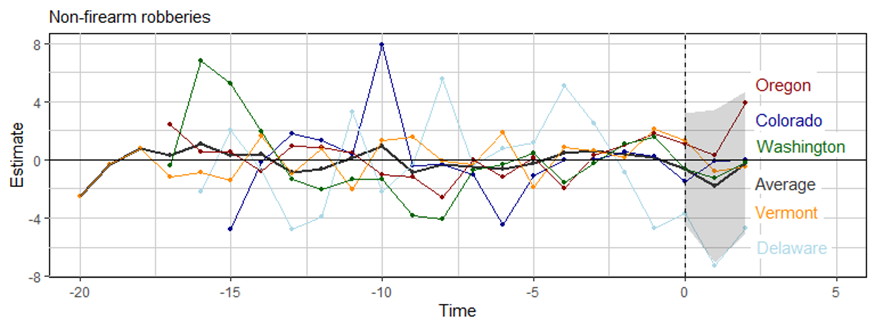


# **Appendix Figure 6. Knife Assaults per 100,000 Persons Relative to Synthetic Control**


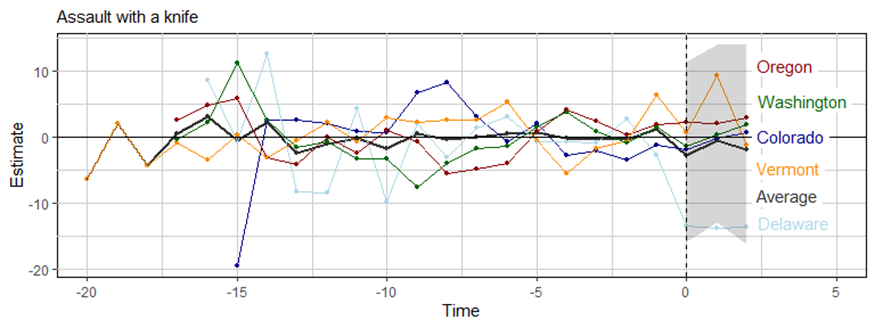


| **Appendix Table 2. Multi-State ASCM with Population Included as a Covariate** | | | | | | |  |
| --- | --- | --- | --- | --- | --- | --- | --- |
|  | **Colorado**  **ATT**  **(p-value)** | **Delaware**  **ATT**  **(p-value)** | **Oregon**  **ATT**  **(p-value)** | **Vermont**  **ATT**  **(p-value)** | **Washington**  **ATT**  **(p-value)** | **Average** **ATT**  **(p-value)** | |
| **Robbery with a firearm** | 3.396 (0.504) | -1.109 (0.889) | -1.451 (0.737) | 2.274 (0.883) | -1.752 (0.717) | 0.272 (0.95) | |
| **Non-firearm robberies** | -1.09 (0.699) | -5.976 (0.411) | 1.147 (0.69) | -0.112 (0.979) | -0.628 (0.773) | -1.332 (0.533) | |
| **Assault with a firearm** | 6.284 (0.586) | 1.036 (0.913) | -6.443 (0.623) | 2.789 (0.875) | -2.318 (0.745) | 0.27 (0.96) | |
| **Assault with a knife** | -0.473 (0.947) | -14.111 (0.65) | 2.84 (0.611) | 3.995 (0.738) | 0.017 (0.996) | -1.547 (0.817) | |

ATT=Average treatment effect on the treated, shown as the number of crimes per 100,000 persons relative to the estimated number of crimes in the synthetic control. P-value in parentheses are generated using the bootstrap procedure.

| **Appendix Table 3. Multi-State ASCM with 10 Years Included in the Pre-Intervention Period** | | | | | | |  |
| --- | --- | --- | --- | --- | --- | --- | --- |
|  | **Colorado**  **ATT**  **(p-value)** | **Delaware**  **ATT**  **(p-value)** | **Oregon**  **ATT**  **(p-value)** | **Vermont**  **ATT**  **(p-value)** | **Washington**  **ATT**  **(p-value)** | **Average** **ATT**  **(p-value)** | |
| **Robbery with a firearm** | 1.417 (0.788) | 3.909 (0.717) | -2.416 (0.581) | 4.897 (0.757) | -0.456 (0.911) | 1.47 (0.759) | |
| **Non-firearm robberies** | 0.786 (0.789) | -3.981 (0.506) | 0.149 (0.963) | -0.435 (0.917) | -0.362 (0.88) | -0.769 (0.719) | |
| **Assault with a firearm** | 8.746 (0.478) | -0.708 (0.946) | -5.092 (0.691) | 4.226 (0.815) | -2.934 (0.687) | 0.847 (0.879) | |
| **Assault with a knife** | 1.926 (0.769) | -15.499 (0.618) | -1.046 (0.842) | 7.259 (0.567) | -3.067 (0.447) | -2.085 (0.746) | |

ATT=Average treatment effect on the treated, shown as the number of crimes per 100,000 persons relative to the estimated number of crimes in the synthetic control. P-value in parentheses are generated using the bootstrap procedure.

| **Appendix Table 4. Multi-State ASCM Restricting to States Without Stand Your Ground or Shall Issue Law Changes During Key Periods** | | | | | | |  |
| --- | --- | --- | --- | --- | --- | --- | --- |
|  | **Colorado**  **ATT**  **(p-value)** | **Delaware**  **ATT**  **(p-value)** | **Oregon**  **ATT**  **(p-value)** | **Vermont**  **ATT**  **(p-value)** | **Washington**  **ATT**  **(p-value)** | **Average** **ATT**  **(p-value)** | |
| **Robbery with a firearm** | -0.196 (0.973) | 2.775 (0.733) | 0.941 (0.843) | 2.793 (0.892) | 1.935 (0.739) | 1.65 (0.749) | |
| **Non-firearm robberies** | -0.478 (0.846) | -3.862 (0.455) | -0.176 (0.966) | -0.485 (0.935) | -0.573 (0.857) | -1.115 (0.683) | |
| **Assault with a firearm** | 9.161 (0.435) | 8.548 (0.348) | -11.312 (0.469) | -2.421 (0.908) | -6.195 (0.493) | -0.444 (0.939) | |
| **Assault with a knife** | 3.903 (0.672) | -15.151 (0.592) | -0.532 (0.945) | 4.849 (0.718) | -3.262 (0.487) | -2.039 (0.751) | |

ATT=Average treatment effect on the treated, shown as the number of crimes per 100,000 persons relative to the estimated number of crimes in the synthetic control. P-value in parentheses are generated using the bootstrap procedure. Excluded states: Idaho, Kansas, Maine, Mississippi, North Dakota, West Virginia, Wisconsin, Wyoming. These states changed Stand Your Ground or Shall Issue laws during the two years prior to CBC implementation or during the follow-up period.

| **Appendix Table 5. Multi-State ASCM Excluding 2020 Data** | | | | | |  |
| --- | --- | --- | --- | --- | --- | --- |
|  | **Colorado**  **ATT**  **(p-value)** | **Delaware**  **ATT**  **(p-value)** | **Oregon**  **ATT**  **(p-value)** | **Vermont**  **ATT**  **(p-value)** | **Washington**  **ATT**  **(p-value)** | **Average** **ATT**  **(p-value)** |
| **Robbery with a firearm** | 1.021 (0.773) | -3.359 (0.724) | -0.566 (0.897) | 2.769 (0.855) | -1.143 (0.782) | -0.256 (0.954) |
| **Non-firearm robberies** | -0.799 (0.786) | -5.478 (0.433) | 0.721 (0.793) | 0.284 (0.937) | -0.955 (0.638) | -1.245 (0.562) |
| **Assault with a firearm** | 5.941 (0.591) | -2.32 (0.798) | -5.256 (0.659) | 3.948 (0.672) | -2.36 (0.724) | -0.009 (0.998) |
| **Assault with a knife** | -0.934 (0.884) | -13.504 (0.651) | 2.193 (0.646) | 4.986 (0.7) | -0.449 (0.923) | -1.542 (0.816) |

ATT=Average treatment effect on the treated, shown as the number of crimes per 100,000 persons relative to the estimated number of crimes in the synthetic control. P-value in parentheses are generated using the bootstrap procedure.

| **Appendix Table 6. Multi-State ASCM Excluding States with Substantial Missing Data** | | | | | |  |
| --- | --- | --- | --- | --- | --- | --- |
|  | **Colorado**  **ATT**  **(p-value)** | **Delaware**  **ATT**  **(p-value)** | **Oregon**  **ATT**  **(p-value)** | **Vermont**  **ATT**  **(p-value)** | **Washington**  **ATT**  **(p-value)** | **Average** **ATT**  **(p-value)** |
| **Robbery with a firearm** | 1.259 (0.822) | -5.676 (0.535) | -0.491 (0.933) | 3.79 (0.822) | -1.412 (0.805) | -0.506 (0.916) |
| **Non-firearm robberies** | 0.125 (0.954) | -5.517 (0.393) | 2.405 (0.558) | -0.931 (0.85) | -0.345 (0.89) | -0.852 (0.704) |
| **Assault with a firearm** | 4.184 (0.713) | 0.807 (0.94) | -4.64 (0.689) | -1.497 (0.933) | -0.711 (0.908) | -0.371 (0.948) |
| **Assault with a knife** | 0.809 (0.931) | -15.564 (0.611) | 2.555 (0.704) | -0.691 (0.963) | 0.399 (0.925) | -2.498 (0.734) |

ATT=Average treatment effect on the treated, shown as the number of crimes per 100,000 persons relative to the estimated number of crimes in the synthetic control. P-value in parentheses are generated using the bootstrap procedure. Excluded states: (Mississippi, Kentucky, New Hampshire, Kansas, and Indiana.

# **Appendix B. Final Regression Model Equation**

The final regression formula took the following form:

𝐶𝑟𝑖𝑚𝑒 𝑐𝑜𝑢𝑛𝑡 (𝑌) ~𝑁𝑒𝑔𝑎𝑡𝑖𝑣𝑒 𝐵𝑖𝑛𝑜𝑚𝑖𝑎𝑙(𝜇,𝜃) 

$$\ln\left( \mu_{i} \right)= \beta_{0}+\sum_{k=1}^{24} \beta_{k}X_{ki}+\beta_{ar1}\ln\left( {lag}_{i} \right)+\ln\left( \frac{{population}_{i}}{1E^{5}} \right)+\varepsilon$$

where the log count of the outcome variable, $\mu_{i},$ (firearm robbery or assault with a firearm) was modeled as a function of 24 covariates ($X_{ki}$), the log of the one-year lagged outcome ($\ln\left( {lag}_{i} \right))$, and a population offset ($\ln\left( \frac{{population}_{i}}{1E^{5}} \right))$. Policy implementation or repeal variables were change-coded following the recommendation of a RAND Corp publication (Schell, Griffin, Morral, 2018). These variables included changes in Stand Your Ground laws, Concealed Carry laws, and CBC laws in the five treated states. Demographic, socioeconomic, criminal justice, and alcohol consumption measures described in more detail in the methods section of the main manuscript were included as annual measures.

*References*

Schell TL, Griffin BA, Morral AR. (2018). Evaluating methods to estimate the effect of state laws on firearm deaths: A simulation study: RAND Corporation.

# **Appendix Figure 7. False Discovery Rates for Four Regression Models Tested**


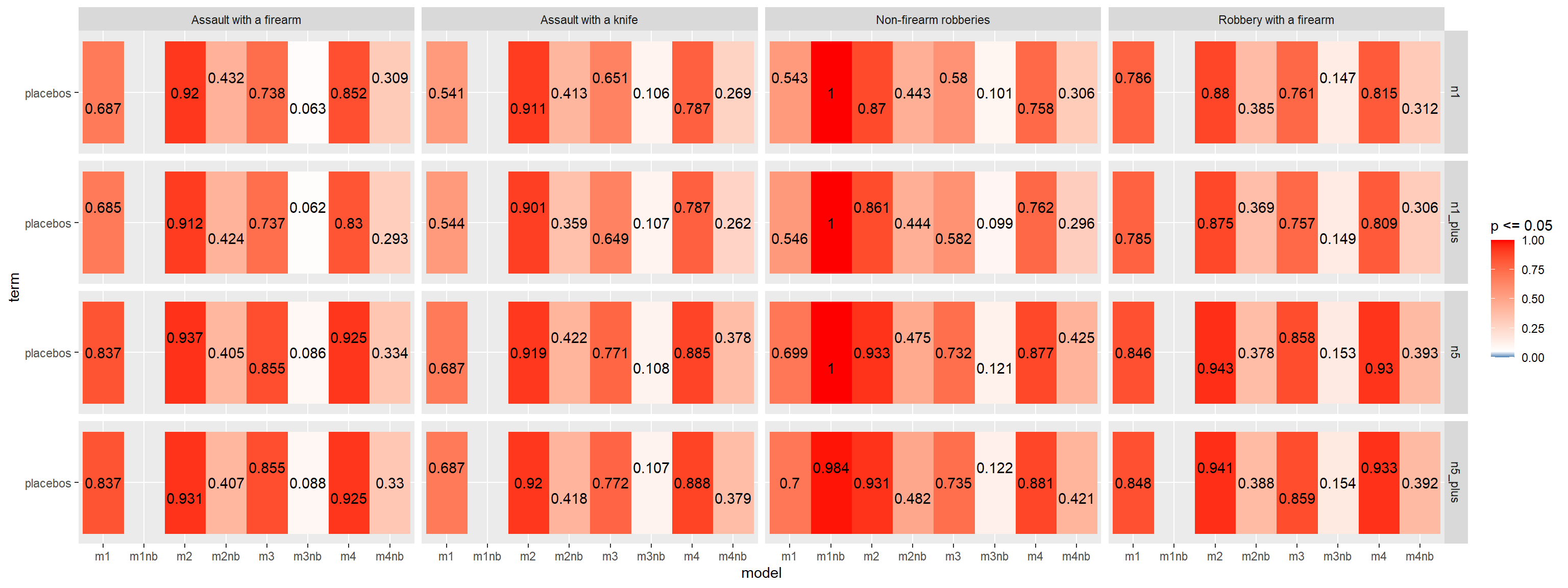


# **Appendix Table 7. Coefficients and P-Values from the Negative Binomial Regression Model**

|  | Colorado  Coef. (p-value) | Delaware  Coef. (p-value) | Oregon  Coef. (p-value) | Vermont  Coef. (p-value) | Washington  Coef. (p-value) |
| --- | --- | --- | --- | --- | --- |
| Robbery with a firearm | -0.30 (0.479) | -0.16 (0.717) | -0.33 (0.445) | -1.572 (0.012) | 0.01 (0.979) |
| Non-firearm robberies | -0.14 (0.644) | -0.15 (0.661) | 0.26 (0.416) | -1.246 (0.020) | -0.04 (0.895) |
| Assault with a firearm | -0.02 (0.962) | -0.04 (0.914) | -0.12 (0.718) | 0.21 (0.640) | 0.12 (0.733) |
| Assault with a knife | 0.02 (0.935) | -0.30 (0.208) | 0.06 (0.802) | -0.58 (0.061) | -0.22 (0.310) |

Results from the regression model described in Appendix B.
